# Deubiquitinase USP18 promotes the progression of pancreatic cancer via enhancing the Notch1-c-Myc axis

**DOI:** 10.18632/aging.103760

**Published:** 2020-10-13

**Authors:** Long Feng, Kai Wang, Ping Tang, Suyun Chen, Tiande Liu, Jun Lei, Rongfa Yuan, Zhigang Hu, Wen Li, Xin Yu

**Affiliations:** 1 Department of Oncology, The Second Affiliated Hospital of Nanchang University, Nanchang University, Nanchang, China; 2 Hepatopancreatobiliary Surgery Division, Department of General Surgery, The Second Affiliated Hospital of Nanchang University, Nanchang University, Nanchang, China; 3 Department of General Surgery, Hunan Youxian People's Hospital, Youxian, China; 4 The Second Clinical Medical College, Nanchang University, Nanchang, China

**Keywords:** USP18, pancreatic cancer, Notch1, c-Myc, ubiquitination

## Abstract

The dysregulation of deubiquitinating enzymes (DUBs), which regulate the stability of most cellular proteins, has been implicated in many human diseases, including cancers. Thus, DUBs can be considered potential therapeutic targets for many cancers. However, the role of deubiquitinase ubiquitin-specific protease 18 (USP18) in pancreatic cancer remains unknown. Here, we found that the deubiquitinase ubiquitin-specific protease 18 (USP18) is significantly upregulated in pancreatic cancer and is correlated with a shorter median overall and relapse-free survival. A functional assay demonstrated that overexpression of USP18 resulted in increased proliferation of pancreatic cancer cells. Conversely, these phenomena were reversed after USP18 was silenced in pancreatic cancer cells. Further investigation revealed that USP18 promoted cell progression by increasing c-Myc expression, which has been reported to control pancreatic cancer progression, and our data demonstrated that c-Myc is key for USP18-mediated pancreatic cancer cell progression *in vitro* and *in vivo*. Moreover, we found that USP18 promoted pancreatic cancer progression via upregulation of Notch-1-dependent c-Myc. Mechanistically, USP18 interacts with and removes K48-linked ubiquitin chains from Notch1, thereby stabilizing Notch1 and promoting the Notch1-c-Myc pathway. Our work identifies and validates USP18 as a pancreatic cancer oncogene and provides a potential druggable target for this intractable disease.

## INTRODUCTION

Pancreatic cancer is an aggressive malignant disease with a poor prognosis, and its death rate is almost equal to its incidence [1]. Pancreatic cancer is a deadly disease with an estimated 5-year survival rate of less than 5% worldwide [2]. Despite progress in treatment strategies, the decrease in mortality rate for this disease is still slow due to its aggressive growth and difficulties in early diagnosis [3]. Therefore, the exploration of diagnostic molecular biomarkers and targets with high therapeutic efficacy is urgently needed to improve the prognosis of pancreatic cancer patients.

Post-translational modification by deubiquitination could modulate functions of target proteins, the fate of proteins and their intracellular mechanisms [4]. Deubiquitination is the reverse process of ubiquitination and is mediated by a group of proteins called deubiquitinating enzymes (DUBs) [5]. Deubiquitinating enzymes, which counteract ubiquitination by cleaving poly- or mono-ubiquitin from target proteins, also play essential roles in various physiological processes [6]. For example, USP4 interacts with and deubiquitinates TCF4, which inhibits β-catenin-dependent transcription [7]. Another DUB, USP49, negatively regulates cellular antiviral responses via deconjugation of K63-linked ubiquitination of MITA [8]. Thus far, many studies have found that various DUBs serve as tumour suppressors or oncogenes depending on their regulation of multiple biological processes [9, 10]. Zhou et al. found that USP4, as a potential novel oncogene, promotes Glioblastoma multiforme (GBM) by activating the ERK pathway through TGF-β regulation [11]. However, the precise role and underlying signalling cascade of DUBs in pancreatic cancer progression remain unclear.

Recently, Ubiquitin-specific peptidase (USP) 18 has been shown to be involved in tumorigenesis [12–14]. USP18 belongs to the USP subfamily of DUBs and mediates deubiquitination of target proteins [15–17]. Ubiquitin-specific peptidase (USP) 18 is known as an interferon (IFN)-stimulated gene 15 (ISG15) isopeptidase and a negative regulator of type I and type III IFN signalling [12, 13]. Previous studies have reported that USP18 has an important role in tumorigenesis [18, 19]. For instance, downregulation of USP18 reduces acute promyelocytic leukaemia cell growth and induces apoptosis, whereas silencing USP18 in glioblastoma cells enhances IFN-induced apoptosis [20]. Tan et al. have demonstrated that USP18 promotes breast cancer growth by upregulating EGFR and activating the AKT/Skp2 pathway. These studies have suggested that USP18 may play an important role in the tumorigenesis and progression of cancer. Importantly, we observed elevated USP18 mRNA in pancreatic cancer tissues though the Cancer Genome Atlas (TCGA) dataset, which was analysed using GEPIA software (http://gepia.cancer-pku.cn/) (Supplementary Figure 1). These studies suggest that USP18 may play an important role in pancreatic cancer tumorigenesis and development. However, its specific mechanism of action in the process of pancreatic cancer progression is still unclear.

In the present study, we aimed to investigate the prognostic values and biological functions of USP18 and its associated molecular mechanisms in pancreatic cancer. We first evaluated the expression level of USP18 in pancreatic cancer tissues and investigated the association of the USP18 expression level with clinicopathological features and patient survival. Then, we studied the role of USP18 in the progression of pancreatic cancer in both *in vivo* and *in vitro* models. Finally, we explored the effect of USP18 on cell signalling pathways in pancreatic cancer cells.

## RESULTS

### Increased USP18 expression is correlated with tumour progression and poor prognosis in pancreatic cancer patients

**Figure 1:**
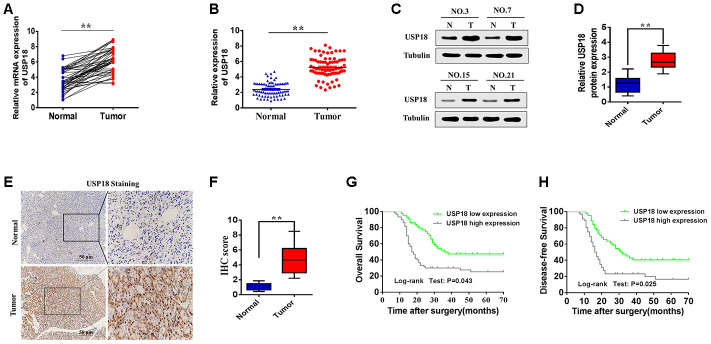
**Relative USP18 expression in pancreatic cancer and its clinical significance.** (**A**, **B**) Determination (**A**) and quantification (**B**) of USP18 mRNA levels in pancreatic cancer tissues and in paired normal tissues by qRT-PCR analysis. Tubulin was used as a loading control. ^**^p<0.01. (**C** and **D**) Relative expression of USP18 in pancreatic cancer tissues and adjacent non-tumour tissues by western blotting analysis. Statistical differences were analysed using the paired t test. ^**^p<0.01. (**E** and **F**) Representative images (**E**) and quantification (**F**) of USP18 staining in paired pancreatic cancer tissues. Scale bar, 50 μm. (**G** and **H**) Relationship between USP18 expression with poor overall survival and disease-free survival in pancreatic cancer patients.

To determine the role of USP18 in the aggressiveness of pancreatic cancer, we firstly examined USP18 expression in pancreatic cancer tissues and corresponding adjacent tissues by qRT-PCR. As shown in Figure 1A, 1B, qRT-PCR revealed that the average fold change of USP18 mRNA expression in pancreatic cancer tissues compared with adjacent nontumor tissues. Next, we further examined the expression levels of USP18 in the pancreatic cancer tissues using Western blot. The results showed that USP18 protein was upregulated in pancreatic cancer tissues compared with adjacent nontumour tissues (Figure 1C, 1D). Moreover, USP18 expression was analysed in 108 pancreatic cancer tissue samples and was compared with the expression in adjacent nontumour tissues by immunohistochemical staining. The pancreatic cancer tissues exhibited greater immunoreactivity, whereas the normal gastric tissues exhibited less immunoreactivity (Figure 1E). The quantitative scoring showed that USP18 protein was expressed at significantly higher levels in pancreatic cancer tissues compared with adjacent nontumour tissues (Figure 1F). These results indicated that the expression of USP18 is significantly upregulated in pancreatic cancer tissues. In addition, we have also detected the expression level of ISG15, which is the product of interferon (IFN)-stimulated gene 15. As shown in Supplementary Figure 2, the expression of ISG15 is significantly upregulated in pancreatic cancer tissues.

Next, we analysed the relationship between USP18 expression and clinicopathological factors in 108 pancreatic cancer patients (Table 1). No significant association was observed between USP18 expression and age or histological type, but USP18 expression was significantly correlated with tumour size(p=0.004) and tumour stage (p<0.001). Additionally, to further explore the efficiency of USP18 in the survival of pancreatic cancer patients, high USP18 expression in pancreatic cancer patients was correlated with poor Overall Survival and Disease-Free Survival (Figure 1G and 1H). Multivariate Cox regression analysis further revealed that high USP18 expression was an independent predictor of poor survival in patients with pancreatic cancer (Table 2). Collectively, these results suggested that USP18 may play a role in pancreatic cancer development and progression.

**Table 1:** Relationship between USP18 expression and clinicopathological features.

**Parameters**	**n**	**USP18 expression**		**P value**
		**Low(n=45)**	**High(n=63)**	
**Age (years)**				*P=0.841*
≤65	42	18	24	
>65	66	27	39	
**Sex**				*P=0.579*
Female	49	19	30	
Male	59	26	33	
**Tumor size(cm)**				*P=0.004*
<5	75	38	37	
≥5	33	7	26	
**TNM stage**				*P<0.001*
T1-T2	48	36	12	
T3-T4	60	9	51	
**Distant metastasis**				*P=0.276*
No	78	35	43	
YES	30	10	20	
**Lymph node status**				*P=0.501*
No	40	15	25	
YES	68	30	38	
**Histologic grade**				*P=0.828*
High	30	12	18	
Low to medium	78	33	45	
**Differentiation**				*P<0.0001*
Well	37	30	7	
Moderate/Poor	71	15	56	

**Table 2:** Univariate and multivariate analyses of overall survival in PC patients.

**Parameters**	**Univariate analysis**			**Multivariate analysis**		
	**HR**	**95%CI**	***P* value**	**HR**	**95%CI**	***P* value**
**Age**	0.789	0.235-2.313	0.472	—	—	—
(≥65 vs <65)						
**Sex**	1.382	0.516-3.636	0.673	—	—	—
(Female vs Male)						
**Distant metastasis**	0.936	0.428-3.528	0.731	—	—	—
(No vs Yes)						
**Lymph node status**	1.647	0.741-4.273	0.526	—	—	—
(No vs Yes)						
						
**Histologic grade**	0.786	0.412-2.522	0.429	—	—	—
(High vs Low to medium)						
						
**Tumor size**	1.637	1.145-4.176	0.002	1.412	1.031-3.629	0.017
(<5 vs ≥6)						
**TNM stage**	3.176	1.776-4.361	0.024	2.134	1.412-4.062	0.033
(T1-T2 vs T3-T4)						
**Differentiation**	1.462	1.162-2.863	0.007	1.235	0.814-2.431	0.037
(Well vs Moderate/Poor)						
**USP18 expression**	2.874	1.451-4.524	0.001	1.662	1.214-3.647	0.007
(High vs Low)						

### USP18 promotes PC cell growth *in vitro* and *in vivo*

**Figure 2:**
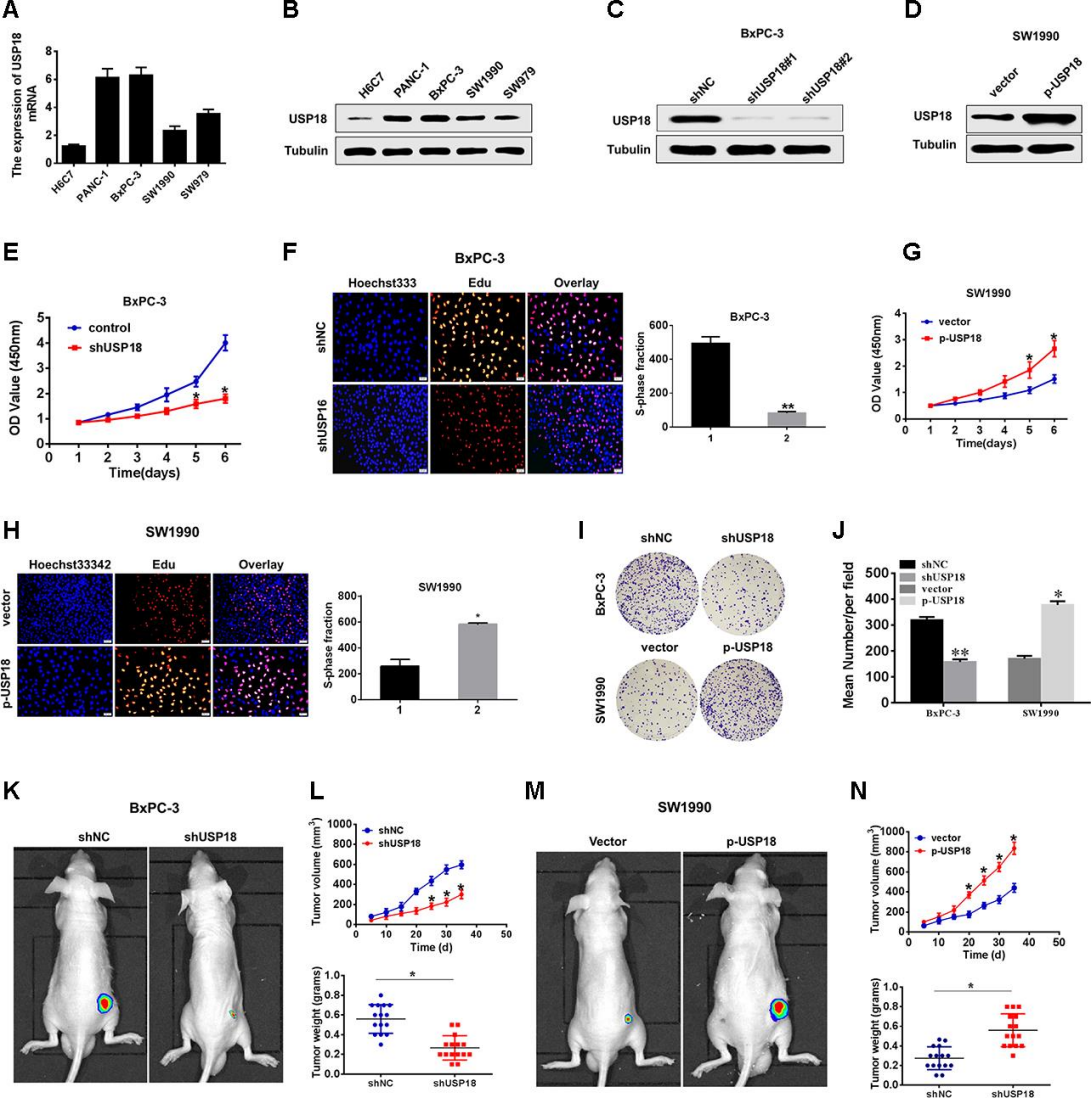
**USP18 promotes pancreatic tumour growth *in vivo* and *in vitro.*** (**A**, **B**) USP18 mRNA and protein levels in pancreatic cells and the immortalized H6C7 line. (**C** and **D**) USP18 protein levels in BxPC-3 and SW1990 cells after transfection with shUSP18 or p-USP18. (**E**) CCK-8 assay showing proliferation of pancreatic cancer cells following USP18 knockdown. ^*^p<0.05. (**F**) Proliferation capacities were detected by EdU assays in BxPC-3 cells transfected with the shNC or the shUSP18 plasmid ^*^p<0.05. (**G** and **H**) CCK-8 and EdU assay showing proliferation of pancreatic cancer cells transfected with the vector or the p-USP18 plasmid. (**I** and **J**) Colony-forming assays showing the cell viability of pancreatic cancer cells transfected with the indicated plasmid (*p<0.05, **p<0.01). (**K**–**N**) BxPC-3/shUSP18 cells or SW1990/p-USP18 were subcutaneously injected into nude mice. Tumour volumes were measured on the indicated days. At the experimental endpoint, tumours were dissected, photographed and weighed (n=15, *p<0.05).

To investigate the potential biological function of USP18 in pancreatic cancer development, we first determined USP18 expression in pancreatic cancer cell lines. The qRT-PCR and Western blot results showed that USP18 was significantly upregulated in PC cells compared with the normal epithelial cell line H6C7 (Figure 2A, 2B). Then, we silenced USP18 expression in BxPC-3 cells by transfection with USP18 shRNA. As shown in Figure 2C, 2D, BxPC-3 cells transfected with USP18 shRNA exhibited an obviously decreased USP18 protein expression level compared with the control group, and SW1990 cells transfected with p-USP18 exhibited an obviously increased USP18 protein expression level compared with the vector. Furthermore, we detected the effects of USP18 on pancreatic cancer cell proliferation by CCK8 and EdU assay. The CCK8 and EdU assay data showed that silencing USP18 obviously suppressed pancreatic cancer cell growth *in vitro* (Figure 2E, 2F). In contrast, USP18-overexpressing cells showed a significantly higher *in vitro* proliferation rate than control cells (Figure 2G–2H). Consistent with these findings, Colony-forming assays also showed that USP18 knockdown decreased the cell viability of BxPC-3 cells, whereas overexpression of USP18 increased the cell viability of SW1990 cells (Figure 2I and 2J).

Next, we then subjected the USP18-knockdown or USP18-overexpressing cells to tumour xenograft experiments. After 5 weeks of growth, USP18-knockdown BxPC-3 cells (BxPC-3/shUSP18) exhibited reduced tumour growth in nude mice, whereas USP18-overexpressing SW1990 cells (SW900/p-USP18) exhibited significantly greater tumour growth compared with their respective controls. Consistent with this, the average tumour weight in mice bearing BxPC-3/shUSP18 cells significantly decreased, whereas that in mice bearing SW900/p-USP18 cells obviously increased, when compared to those of the respective controls (Figure 2K–2N). In conclusion, these data collectively indicated that USP18 contributes to growth of pancreatic cancer cells *in vitro* and *in vivo*.

### USP18 promotes PC cell growth by facilitating cell cycle progression

**Figure 3:**
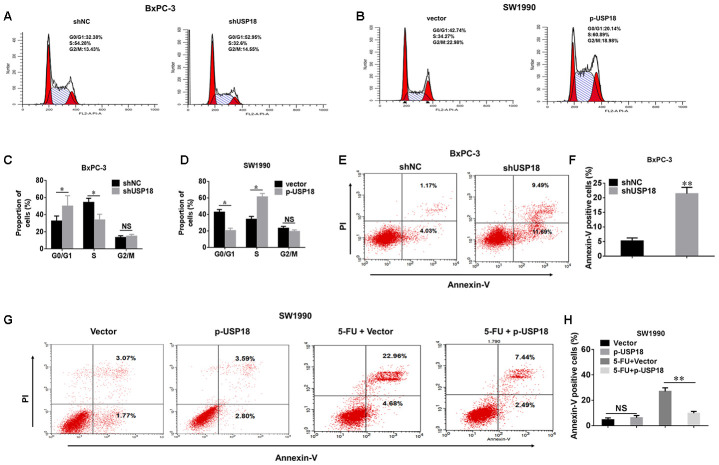
**The effects of USP18 on pancreatic cancer cell growth.** (**A**–**D**) Detection of cell cycle stage in BxPC-3/shUSP18 or SW1990/p-USP18 cells. Results are expressed as a peak diagram (**A**, **B**) and the calculated distribution of cells in G0/G1, S, and G2/M phases (**C**, **D**). *p<0.05. (**E**–**H**) Results are expressed as a scatter diagram and the calculated percentage of the annexin-V-positive cell population in different groups (^*^p<0.05).

To further understand the mechanism by which USP18 contributes to PC cell growth, we investigated the effects of USP18 knockdown on the cell cycle and apoptosis. Compared with the control group, USP18 shRNA treatment led to a significant accumulation of cells in G0/G1 phase, along with a decrease in the number of cells in S-phase. And we analyzed changes of the cell cycle in USP18-overexpressing cells. USP18 overexpression was associated with an increase of cells entering S phase and a corresponding decline of cells in the G0/G1 phase (Figure 3A–3D). This conclusion was further confirmed by examining cell cyclerelated proteins. Expression of cyclin D1, CDK4 and CDK6 was significantly upregulated in USP18-overexpressing cells, whereas expression of these proteins was downregulated in USP18-knockdown cells (Supplementary Figure 3). Furthermore, as shown in Figure 3E, 3F, the percentage of apoptotic cells was significantly increased in the USP18 knockdown group compared with the control group. Moreover, when treated with 5-fluorouracil (100 μM for 36 h) in these indicated cells, the percentage of apoptotic cells was significantly decreased in USP18-overexpressing cells (SW1990/p-USP18) cells compared with the control group (Figure 3G–3H). Overall, these results suggested that USP18 promotes pancreatic cancer cell proliferation by facilitating cell cycle progression and inhibiting cell apoptosis.

### Stable knockdown of USP18 represses c-Myc expression in PC cells

**Figure 4:**
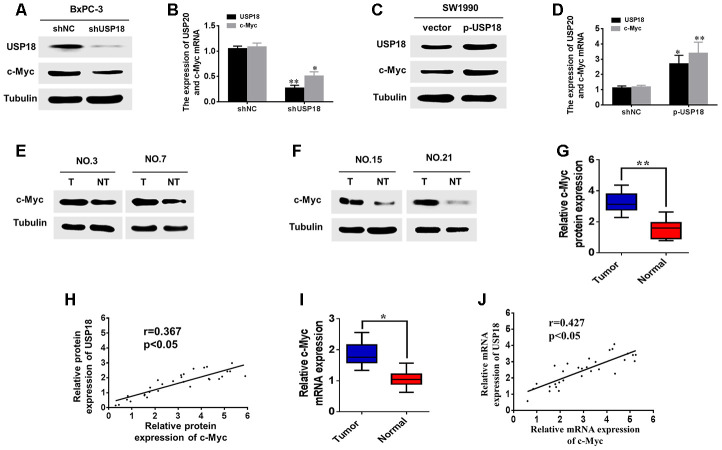
**USP18 promotes pancreatic cancer cell growth by upregulating c-Myc expression.** (**A**, **B**) analyses were performed to detect the USP18 and C-Myc expression levels in BxPC-3 cells transfected with shUSP18 or shNC. (**C** and **D**) Western blot and qRT-PCR analyses were performed to detect the USP18 and C-Myc expression levels in SW1990 cells transfected with p-USP18 or vector. (**E**–**G**) Determination and quantification of c-Myc protein levels in pancreatic cancer tissues and paired non-tumour tissues by western blotting. Tubulin served as a loading control. (**H**) Scatter plots of USP18 and c-Myc protein expression in pancreatic cancer. (**I**) Determination of c-Myc mRNA levels in pancreatic cancer tissues and paired non-tumour tissues by qRT-PCR. (**J**) Scatter plots of USP18 and c-Myc mRNA expression in pancreatic cancer.

Previous studies have demonstrated that c-Myc is a key regulator of pancreatic cancer cell proliferation, and RNA-seq data have indicated that c-Myc is down-regulated in USP18-knockdown cells (Supplementary Table 1). To further determine whether USP18 could regulate c-Myc expression, we first measured the protein and mRNA levels of c-Myc in USP18-knockdown pancreatic cancer cells. The result showed that downregulation of USP18 significantly decreased c-Myc mRNA and protein levels in BxPC-3 cells (Figure 4A, 4B). Conversely, overexpression of USP18 markedly increased the protein and mRNA levels of c-Myc in SW1990 cells (Figure 4C and 4D).

Next, using Western blot, we measured the protein levels of c-Myc in 30 pancreatic cancer tissues with high USP18 expression. Our results indicated that protein levels of c-Myc were significantly upregulated in pancreatic cancer tissues compared with corresponding adjacent normal tissues (Figure 4E–4G). Scatter plots revealed that USP18 and c-Myc protein expression levels were positively correlated in pancreatic cancer tissues (Figure 4H). In addition, qRT-PCR also showed that the c-Myc mRNA levels were significantly upregulated in pancreatic cancer tissues compared with corresponding adjacent normal tissues (Figure 4I), consistent with the western blotting results. Furthermore, scatter plots showed that the USP18 and c-Myc mRNA expression levels were positively correlated in pancreatic cancer tissues (Figure 4J). Collectively, these data strongly suggested that USP18 positively regulates c-Myc in PC.

### c-Myc is crucial for USP18-mediated pancreatic cancer progression

**Figure 5:**
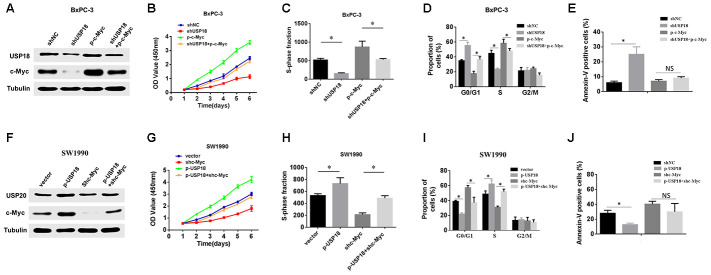
**c-Myc is required for USP18-mediated pancreatic cancer growth.** (**A**) Overexpression of ectopic c-Myc attenuated the loss of c-Myc expression in BxPC-3/shUSP18 cells. (**B**–**D**) CCK-8 assay, Edu assay and flow cytometry show that the overexpression of ectopic c-Myc significantly rescued the decreased cell proliferation ability of BxPC-3/shUSP18 cells, ^*^p<0.05. (**E**) Results are expressed as the calculated percentage of Annexin-V-positive cells. *p<0.05. (**F**) Knockdown of c-Myc expression markedly inhibited the increase in c-Myc expression observed in SW1990/p-USP18 cells. (**G**–**I**) CCK-8 assay, Edu assays and flow cytometry demonstrate that c-Myc inhibition significantly reduced the USP18-enhanced cell proliferation observed in SW1990/p-USP18 cells. (**J**) Results are expressed as the calculated percentage of Annexin-V-positive cells. *p<0.05.

To further validate that USP18 mediated the growth of PC cells by regulating c-Myc, we first increased the expression of c-Myc in USP18 knockdown PC cells and then measured the USP18 and c-Myc protein expression levels and cell proliferation. The western blotting data showed that the overexpression of c-Myc markedly attenuated the loss of c-Myc expression in BxPC-3/shUSP18 cells (Figure 5A). Simultaneously, CCK8 and EdU assays showed that the reduced proliferation induced by USP18 knockdown in BxPC-3 cells was partly abolished by the introduction of c-Myc (Figure 5B–5C). In keeping with these findings, c-Myc overexpression significantly rescued cell cycle arrest in G0/G1 phase induced by USP18 knockdown in PANC-1 cells (Figure 5D). Moreover, flow cytometry revealed that the anti-apoptotic effect of USP18 knockdown could be partially reversed by the introduction of c-Myc in PANC-1 cells (Figure 5E). In contrast, downregulation of c-Myc inhibited the increase in c-Myc expression and significantly reduced cell proliferation in USP18-overexpressing SW1990 cells (Figure 5F and 5G). Consistently, the loss of c-Myc reversed the cell cycle arrest and apoptosis induced by USP18 overexpression in SW1990 cells (Figure 5I–5J). Taken together, these results demonstrated that USP18 regulates c-Myc protein expression to influence PC progression.

### USP18 contributes to the progression of pancreatic cancer through enhancing the Notch1-c-Myc axis

**Figure 6:**
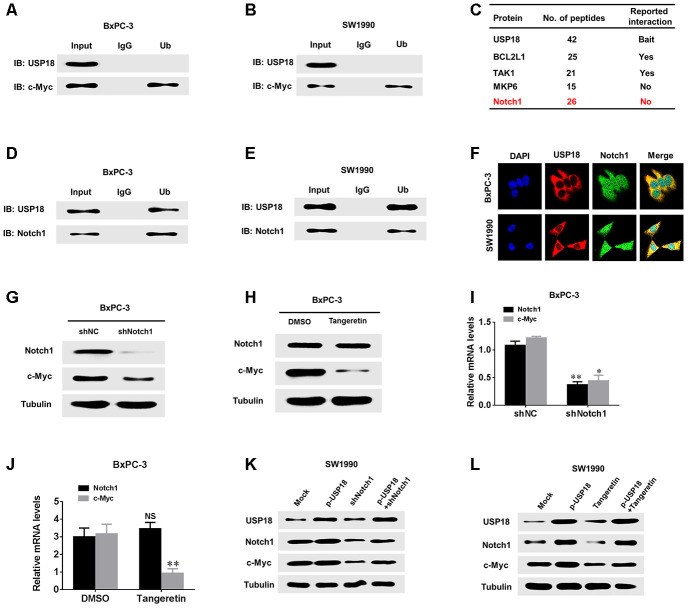
**USP18 regulates c-Myc expression through Notch1.** (**A**, **B**) Co-IP showing that endogenous USP18 and c-Myc in PC cells were not directly bound. (**C**) Mass spectrometry data show that Notch 1 and USP18 are combined. (**D** and **E**) Co-IP showing direct binding of endogenous USP18 and c-Myc in pancreatic cancer cells. (**F**) Co-localization studies of pancreatic cancer cells using an anti-USP18 antibody (1:200, green) and an anti-Notch1 antibody (1:200, red), followed by DAPI nuclear counterstaining (blue). The merged images of USP18 (green) and Notch1 (red) with DAPI (blue) are also shown. (**G** and **I**) Protein and mRNA levels of c-Myc assessed by western blotting and qRT-PCR in pancreatic cancer cells transfected with shNotch1 or shNC. (**H** and **J**) Protein and mRNA levels of c-Myc assessed by western blotting and qRT-PCR in pancreatic cancer cells treated with Notch1 inhibitor (Tangeretin). (**K** and **L**) Protein levels of Notch1 and c-Myc in pancreatic cancer cells transfected with the indicated plasmid or treated with Notch1 inhibitor (Tangeretin) as assessed by western blotting.

USP18 has been reported to interact with different substrates to exert its effects [16–18]. To further clarify the mechanism through which USP18 regulates c-Myc in pancreatic cancer cells, we first determined whether there was a direct interaction between the USP18 and c-Myc proteins. However, co-IP revealed no direct interaction between these proteins (Figure 6A and 6B). Recent studies have shown that c-Myc promotes cancer development by acting as a target of Notch1 to establish a Notch1-c-Myc pathway. Additionally, the mass spectrum data indicate that Notch1 and USP18 are combined (Figure 6C). Furthermore, as shown in Supplementary Figure 4, the expression levels of downstream targets as Hes1 and DTX1 were significantly upregulated in USP18-overexpressing cells, whereas expression of these proteins was downregulated in USP18-knockdown cells. Therefore, we speculated that USP18 regulates c-Myc via Notch1. To test this hypothesis, we first observed whether USP18 and Notch1 directly interact in pancreatic cancer cells, and interestingly, co-IP demonstrated an interaction between USP18 and Notch1 (Figure 6D and 6E). Confocal microscopy was used to further confirm the co-localization of USP18 and Notch1 in pancreatic cancer cells, which provided further evidence of an interaction between these two proteins (Figure 6F). Next, to explore whether Notch1 regulates c-Myc in pancreatic cancer cells, we measured the changes in c-Myc expression in Notch1-knockdown BxPC-3 cells. The results showed that the knockdown of Notch1 or Notch1 inhibitor (Tangeretin) significantly decreased c-Myc protein and mRNA expression in BxPC-3 cells (Figure 6G–6J). These findings demonstrated that Notch1 regulates c-Myc expression in pancreatic cancer cells.

We further aimed to elucidate whether USP18 affects the expression of c-Myc via Notch1. To further verify that USP18 regulates c-Myc expression through Notch1 in pancreatic cancer cells, we decreased the expression of Nothc1 in USP18-overexpressing pancreatic cancer cells and then observed the Nothc1 and c-Myc protein levels. The results showed that the donwnregulation of Notch1 inhibited the increases in c-Myc expression observed in USP18-overexpressing pancreatic cancer cells (Figure 6K–6L). Importantly, we also measured the protein levels of Notch1 in 30 pancreatic cancer tissues with high USP18 expression. Our results indicated that protein levels of Notch1 were significantly upregulated in pancreatic cancer tissues compared with corresponding adjacent normal tissues (Supplementary Figure 5A and 5B). Scatter plots revealed that USP18 and Notch1 protein expression levels were positively correlated in pancreatic cancer tissues (Supplementary Figure 5C). Thus, these results demonstrated that the USP18-mediated regulation of c-Myc-induced pancreatic cancer cell progression is dependent on Notch1.

### USP18 stabilizes and deconjugates K48-linked ubiquitination of Notch1

**Figure 7:**
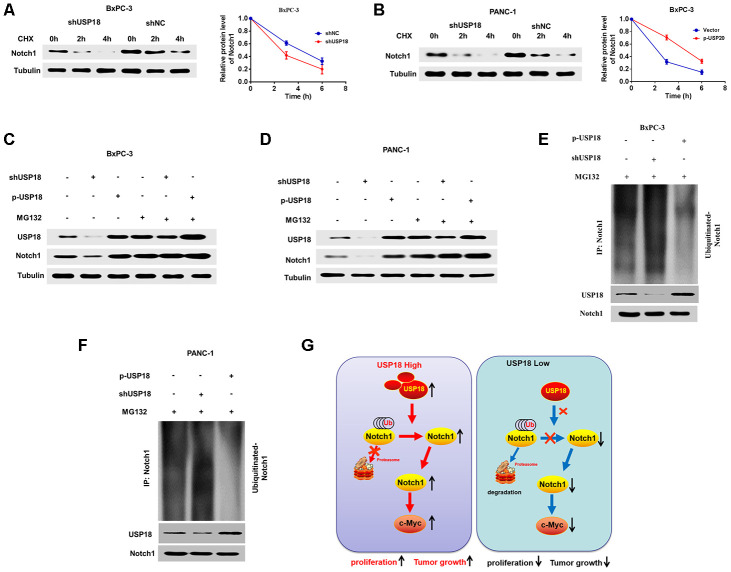
**USP18 stabilizes and deconjugates K48-linked ubiquitination of Notch1.** (**A**, **B**) The Pancreatic cancer cells were exposed to cycloheximide (CHX) (20 μmol/L) for the indicated times, and the degradation of Notch1 was detected with an anti-Notch1 antibody. (**C** and **D**) USP18 had no effect on Notch1 expression, as assessed by MG132 after transfection with shUSP18 or p-USP18 plasmid in pancreatic cancer cells. (**E** and **F**) Knockdown or exogenous expression of USP18 altered the ubiquitination of Notch1. The cells in each group were treated with the proteasomal inhibitor MG132. Cell lysates were prepared and subjected to immunoprecipitation with an anti-Notch1 antibody. The level of ubiquitin binding on Notch1 was detected by western blotting with an anti-Ub-K48 antibody. (**G**) Proposed model by which USP18 promotes PC progression by modifying Notch1/c-Myc axis.

These experiments verified that USP18 could directly bind to Notch1 and regulate c-Myc expression via Notch1. We then further assessed the mechanisms through which USP18 regulates Notch1. Given that knockdown of USP18 leads to increased K48-linked ubiquitination of proteins, we speculated that USP18 K48-linked ubiquitination of Notch1 and thus stabilizes Notch1. Indeed, we found here that USP18 silencing could increase the turnover rate of Notch1 in the presence of cycloheximide (CHX) (Figure 7A and 7B). Furthermore, the Notch1 protein level was not different in USP18-overexpressing or USP18-knockdwon cells following treatment with the proteasome inhibitor MG132 (Figure 7C–7D). Moreover, USP18 overexpression reduced the K48-linked ubiquitination level of Notch1, whereas USP18 silencing significantly increased Notch1 K48-linked ubiquitination (Figure 7E–7F). These data collectively suggested that USP18 rescues Notch1 from proteasome-dependent degradation of by deconjugating K48-linked ubiquitination of Notch1 (G).

## DISCUSSION

Pancreatic cancer is one of the most serious malignant tumours [22]. Unfortunately, pancreatic cancer is usually diagnosed in patients with advanced-stage disease, and less than 18% of patients are eligible for radical resection [23, 24]. Clinical studies have demonstrated that aggressive growth is the most important factor that determines the therapy and contributes to the low survival rate and poor prognosis of pancreatic cancer [25]. Therefore, identification of sensitive and specific markers to improve the diagnosis and to accurately evaluate prognosis remains an urgent issue. In the present study, our results revealed that USP18 is an oncogene in pancreatic cancer and provides a potential druggable target for this intractable disease.

USP18, which belongs to the USP subfamily of DUBs, has been reported to regulate a wide variety of cellular events by mediating the deubiquitination of various target proteins [13, 18, 19]. In addition, independent of its protease activity, USP18 functions as a major negative regulator of the type I interferon response showing that USP18 is - at least - a bifunctional protein. Recently, many studies have found that the expression of USP18 is up-regulated in various tumour tissues and acts as an oncogene. For instance, Tan et al. found that USP18 promotes breast cancer growth by upregulating EGFR and activating the AKT/Skp2 pathway [19]. USP18 was revealed to be upregulated in lung cancer, and reduced USP18 expression was found to be associated with significantly longer cancer-specific survival in patients with muscle-invasive bladder cancer [26, 27]. These findings suggest that USP18 may act as an oncogene in various types of cancer. Therefore, we analysed the TCGA database to further evaluate the expression of USP18 in pancreatic cancer. The results showed that USP18 expression was increased in pancreatic cancer tissues compared with normal tissues. Furthermore, we found that the expression of USP18 was significantly upregulated in pancreatic cancer tissues compared with adjacent normal tissues. Importantly, high USP18 expression in pancreatic cancer patients was correlated with poor overall survival and disease-free survival; this was not observed in patients who had a low level of USP18 expression. Multivariate analyses further revealed that high USP18 expression was an independent predictive factor for poor OS in pancreatic cancer. Both *in vivo* and *in vitro* data demonstrated that USP18 knockdown impaired cell proliferation in pancreatic cancer. In contrast, USP18-overexpressing cells had a significantly higher proliferation rate than control cells. Moreover, flow cytometry showed that downregulation of USP18 significantly arrested the cell cycle in G1 phase in pancreatic cancer cells. In addition, our results showed that the percentage of apoptotic cells was significantly increased in the USP18 knockdown pancreatic cancer cells. Therefore, all these studies suggested that USP18 may represent a novel indicator of poor prognosis in pancreatic cancer and may function as an oncogene in pancreatic cancer progression.

Recently, convincing research has established that Notch1 signalling is an important cell signalling pathway that is critically needed for the maintenance and regulation of the balance among cell proliferation, differentiation, and apoptosis [28, 29]. Many studies revealed that Notch1 signalling is involved in the regulation of cancer [30]. The c-Myc protein, which is encoded by a gene that is a downstream target of Notch1, regulates protein stabilization, promotes cell division and accelerates cell entry into S phase from G0/G1 phase [31]. In addition, c-Myc promotes cell proliferation and inhibits apoptosis in many tumour cells. Previous studies demonstrated that the Notch1-c-Myc signalling pathway plays an important role in pancreatic cancer progression [32, 33]. However, the regulatory mechanisms of the Notch1-c-Myc signalling pathway in pancreatic cancer is still not clear. Here, we revealed a novel mechanism by which USP18 regulates c-Myc expression and found that it is dependent on activation of the Notch1-c-Myc signalling pathway. This conclusion is based on the observations discussed below. First, our results demonstrated that USP18 regulates c-Myc protein expression to influence pancreatic cancer progression. Second, c-Myc is crucial for USP18-mediated pancreatic cancer progression. Importantly, in this study, our results demonstrated for the first time that USP18-mediated regulation of c-Myc-induced pancreatic cancer cell progression is dependent on Notch1. Overall, these results demonstrated that USP18 contributes to the progression of pancreatic cancer and is dependent on the Notch1/c-Myc signalling pathway(Figure 7G).

Finally, we investigated the molecular mechanism by which USP18 regulates Notch1 expression. USP18, which belongs to the USP subfamily of DUBs, has been reported to regulate a wide variety of cellular events by mediating the deubiquitination of various target proteins [32, 33]. In our study, by screening a panel of DUBs, we demonstrated that USP18 interacts with Notch1. In addition, USP18 functions as a deubiquitinase and stabilizes Notch1, which in turn regulates tumorigenesis and cancer progression in a Notch1-dependent manner. This conclusion is based on the observations discussed below. First, USP18 and Notch1 directly interact in pancreatic cancer cells. Second, USP18 overexpression reduces the K48-linked ubiquitination level of Notch1, whereas USP18 silencing significantly increases the Notch1 K48-linked ubiquitination. Furthermore, the protein level of Notch1 was not different in USP18-overexpressing or USP18-knockdown cells following treatment with the proteasome inhibitor MG132. In addition, the data showed that mutations in all Lys sites of Notch1 abolished Notch1 poly-ubiquitination, which shows that USP18 does not regulate Notch1 expression. Interestingly, the prior study indicated that USP18 shows no reactivity toward ubiquitin but specifically deconjugates the interferon-induced Ubl ISG15 [34]. And, Zhang M. et al. have reported that USP18 recruits USP20 to deconjugate K48-linked ubiquitination chains from STING and promotes the stability of STING [15]. However, our data shown that USP18 interacts with and removes K48-linked ubiquitin chains from Notch1, thereby stabilizing Notch1 in PC cells. This controversial results need further study in the future.

In conclusion, these findings may provide novel insight into the cancer-promoting effects of USP18 and its potential clinical application in pancreatic cancer, which suggests that the USP18/Notch1/c-Myc pathway may act as a crucial regulator of pancreatic cancer progression. Our work identifies and validates USP18 as a pancreatic cancer oncogene and provides a potential druggable target for this intractable disease.

## MATERIALS AND METHODS

### Database analysis

Both the clinical data and the RNA-Seq data from the pancreatic cancer patients shown here were acquired from the TCGA pancreatic cancer cohort within the GEPIA data portal: https://portal.gdc.cancer.gov/. In all, the pancreatic cancer samples logged in the TCGA had both clinical data and RNA-Seq data available for analysis. The Edge R package was applied to acquire the RNA expression matrix.

### Patients and sample collection

We obtained 108 pairs of primary pancreatic cancer tumour tissues and corresponding non-cancer tissues from patients undergoing surgical resection in the general surgery department of the Second Affiliated Hospital of Nanchang University. All resected specimens were frozen and stored at -80°C for further analysis. Each patient provided written informed consent. This study was approved by the Ethics and Research Committee of the Second Affiliated Hospital of Nanchang University.

### Cell lines and culture conditions

Human pancreatic cancer cell lines (PANC-1, Rosie, BxPC-3 and SW-979) and the normal epithelial kidney cell line (H6C7) were obtained from the American Type Culture Collection (Rockville, Maryland). Pancreatic cancer cell lines were cultured in Dulbecco’s Modified Eagle’s Medium supplemented with 10% foetal bovine serum and maintained at 37°C in a humid environment containing 5% CO_2_. H6C7 cells were cultured in keratinocyte-free medium (Invitrogen, Carlsbad, CA, USA) supplemented with 10% foetal bovine serum (Gibco, Grand Island, NY, USA) and antibiotics and were maintained at 37°C and 5% CO_2_.

### Real-time quantitative polymerase chain reaction (qRT-PCR)

Total RNA was isolated using TRIzol reagent (Invitrogen, USA) according to the manufacturer’s instructions. Then, RNA was reverse transcribed using a PrimeScript RT Reagent Kit (Invitrogen, USA). For quantitative polymerase chain reaction (PCR) analysis, qPCR was performed using SYBR Premix Ex Taq (TaKaRa, China) according to the manufacturer’s instructions. Real-Time PCR was used to determine the relative USP18 and GAPDH (internal control) mRNA levels. The primers used for the qRT-PCR assay were as follows: USP18 forward, CCCACAGGCTCATAACTAAAGG-3 and reverse AATATGTAACCATGAGGCCCC; and GAPDH forward, 5’-CCACTCCTCCACCTTTGAC-3’ and reverse 5’-ACCCTGTTGCTGTAGCCA-3’ qPCR was performed as follows: 95°C for 10 min, followed. Real-time PCR parameters were 95 °C for 10 s as a pre-denaturing step followed by 40 PCR cycles of 95 °C for 5 s, 60 °C for 30 s, and 72°C for 10 min. All samples were assayed in triplicate.

### Western blot analysis

The western blot analysis was performed routinely. In brief, equal amounts of protein were separated by 10% SDS-PAGE before transfer to nitrocellulose membranes, which were incubated with the primary antibodies anti-USP18 (1:1000, Abcam), anti-c-Myc (1:1000, Santa Cruz), anti-cyclin D1 (1:1000, Santa Cruz), anti-CDK4 (1:1000, CST), anti-CDK6 (1:1000, CST), anti-Notch1(1:1000, Abcam), anti-Hes1 (1:1000, Abcam), anti-DTX1 (1:1000, Santa Cruz) and anti-Tubulin (1:1000, Abcam) at 4 °C overnight. Then, they were blotted for 1 h at room temperature with the help of an appropriate secondary antibody. Thereafter, ECL reagents were used to visualize bands (Pierce, USA) and signals were measured.

### Constructs and plasmids

The RNA duplexes for shRNA-mediated USP18 were synthesized and cloned into pLKO.1-TRC cloning vector by Genepharma Company (Shanghai, China). In addition, the plasmid of USP18 were cloned into pcDNA3, in-frame with an flag epitope tag. In order to obtain a stable USP18 knockdown OR overexpressing cell lines, the shUSP18 or p-USP18 vectors were transfected into PC cells. Then, the transfected cells were selected by incubation with 2μg/ml of puromycin for 2 weeks. And, the expression of USP18 in PC cell lines stably transfected vectors was examined by Western blot.

### Cell growth assays

For cell proliferation assays, multiple cultures of pancreatic cancer cells were plated in 96-well plates at a density of 5.0x10^3^ cells/well. At the indicated time points, viable cells were assessed using Cell Counting Kit-8 (CCK-8) according to the manufacturer’s instructions. For EdU assay, the cell proliferation rate of PC cells was determined by using a Cell-Light EdU DNA Cell Proliferation Kit (Ribobio, Guangzhou, china) and was calculated as the ratio of the number of EdU-positive cells to the number of total cells (1000 cells for 10 fields of view). The percentage of EdU-positive cells was determined for comparison. For the colony formation assay, PC cells (at the density of 1000 cells per well) were seeded into 6-well plates. After 2 weeks, cells were washed with phosphate buffered saline (PBS), stained with 0.1% crystal violet for 15 min.

### Cell apoptosis analysis

An apoptosis detection kit (UK, USA) was used to detect the apoptosis rate of cells according to the manufacturer's instructions. Briefly, 5x10^5^ cells were centrifuged at 1,000 g for 5 minutes and were then suspended in 180 ml of combined buffer, then incubated with 5 L of annexin V-FITC and 5 L of propidium iodide (pi) in the dark at 37°C for 15 minutes. Apoptotic cells were then detected by flow cytometry. Integrated circuit events.

### Cell-cycle analysis

In all, 1×10^6^ cells were trypsinized, washed twice with PBS, and fixed in 1 ml of precooled 70% ethanol. Cells were again washed twice with PBS. Then, the cells were incubated with propidium iodide (Sigma-Aldrich, St. Louis, Missouri, United States) and ribonucleic acid at room temperature for 30 minutes, and then incubated in the dark at 4°C for 30 minutes. The cell suspension was filtered using a 300 μm mesh screen filter, and adherent cells were removed. The deoxyribonucleic acid content was analysed by flow cytometry (Bioscience Development Bureau of San Jose, California, USA), and the number and proportion of cells in G0/G1, S, and G2/M were estimated using software.

### Immunohistochemistry (IHC) and immunofluorescence (IF) assay

Pancreatic cancer and adjacent tissues were fixed, embedded in paraffin, sectioned and deparaffinized. For IHC, the sections were blocked with serum-free protein blocking buffer (Dakota, Gloucester stroop, Denmark) for 30 minutes and then incubated with anti-USP18 (Abcam, 1:180, Cambridge, UK), anti-Notch1 (1:180, Abcam) or anti-c-Myc (1:180, Abcam) antibodies. For medium frequency analysis, the cells (2x10^3^) on the slide were blocked with 5% bovine serum albumin at room temperature and stained overnight with anti-USP18 (1:100, ABCM), anti-Notch1 (1:100, ABCM) or anti-c-Myc (1:100, ABCM) at 4°C followed by incubation with a fluorescent dye-bound secondary antibody (1:180, Invitrogen) with or without a DAPI counterstain.

### Co-immunoprecipitation (Co-IP), in vivo ubiquitination assay and co-IP-MS

Co-IP determination was performed as previously described [21]. For ubiquitination analysis in vivo, pancreatic cancer cells in which UBR18 was knocked down or overexpressed were exposed to MG132 for 4 hours before harvest. Then, the cell lysate was immunoprecipitated with an anti-Notch 1 antibody, and the ubiquitination level of Notch 1 was evaluated with an anti-Ub antibody. In order to detect Notch1 ubiquitination in pancreatic cancer cells, the cells were transfected with hyaluronic acid -Notch1, His-UPS18, and Flag-Ub constructs for 36 hours and further incubated with MG132 (15 μl) for another 4 hours before analysis. For mass spectrometry, Co-IP samples were validated by Western Blot as above and then subjected to SDS-PAGE. Gels were fixed for 30 min in Coomassie fixative (50% methanol, 10% glacial acetic acid), gel lanes excised, and submitted for mass spectrometry. UPLC-MS/MS was completed using nanoACQUITY UPLC columns (Waters) in front of Orbitrap Elite mass spectrometers (Thermo Fisher Scientific).

### Tumourigenicity assay

Pancreatic cancer cells (1×10^6^ in 100 ml PBS) were injected subcutaneously into the flanks of nude mice (male BALB/c-nu/nu, 6–8 weeks old). Tumour formation in nude mice was monitored, and the tumour volume was measured every 5 days. Tumours were harvested and individually weighed after the mice were anesthetized. The data are presented as tumour weight (mean±SD). The animal work was approved by the Ethics Committee for Animal Experiments of the Second Affiliated Hospital of Nanchang University.

### Statistical analysis

Statistical analyses were performed using the SPSS statistical software package (standard version 19.0 PSS, Chicago, IL). Quantitative data obtained from experiments with biological replicates are shown as the mean ± standard deviation. Linear regression and Pearson correlation analysis were performed. Survival analysis was performed using the Kaplan-Meier and log-rank tests. A two-tailed Student’s t-test was used to analyse the quantitative data; P values < 0.05 were considered statistically significant.

## Supplementary Material

Supplementary Figures

Supplementary Table 1
