# Supplementary material for: Deubiquitinase USP18 promotes the progression of pancreatic cancer via enhancing the Notch1-c-Myc axis
Source: Aging (Albany NY). 2020 Oct 13;12(19):19273–92. doi: 10.18632/aging.103760 (PMC7732327; doi:10.18632/aging.103760)
Supplement: Supplementary Figures [file aging-12-103760-s001..pdf]

SUPPLEMENTARY FIGURES

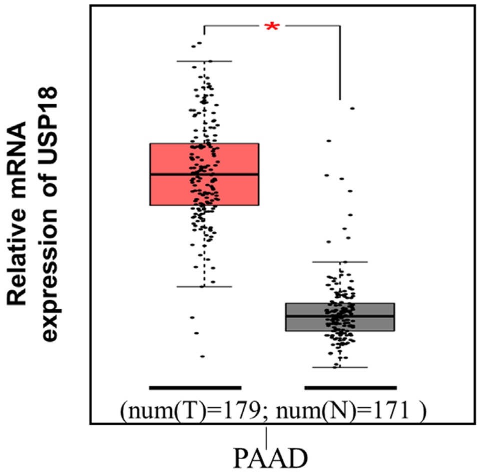

Supplementary Figure 1. The mRNA level of USP18 was upregulated in pancreatic cancer tissues via the Cancer Genome Atlas (TCGA) dataset.

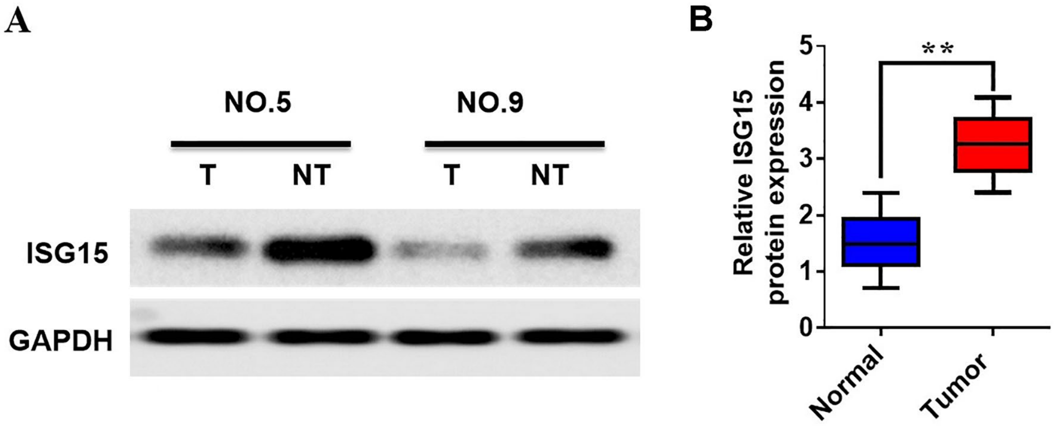

Supplementary Figure 2. (**A**, **B**), Relative expression of ISG15 in pancreatic cancer tissues and adjacent non-tumour tissues by western blotting analysis. Statistical differences were analysed using the paired t test. **p<0.01.

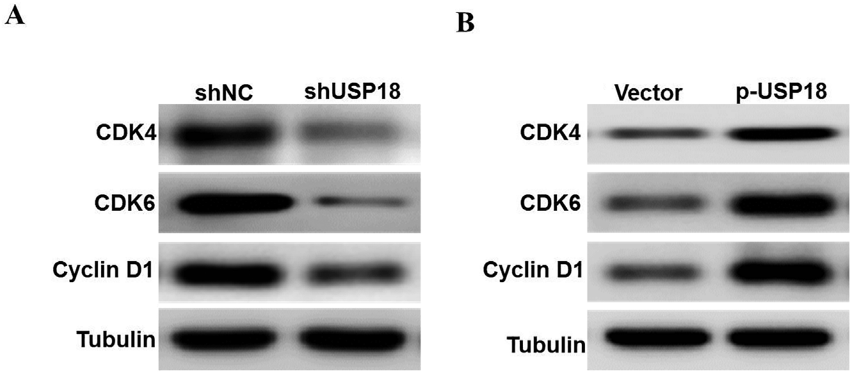

Supplementary Figure 3. (**A**, **B**) Western blot analyses were performed to detect the cyclin D1, CDK4 and CDK6 expression levels in USP18 knockdown or overexpressing cells.

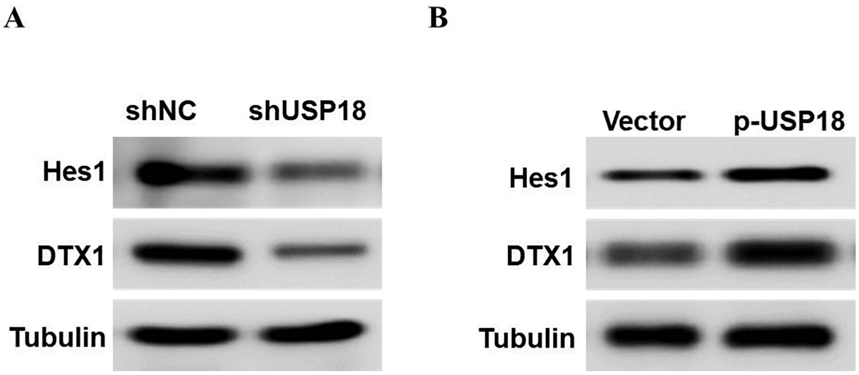

Supplementary Figure 4. (**A**, **B**) Western blot analyses were performed to detect the Hes1 and DTX1 expression levels in USP18 knockdown or overexpressing cells.

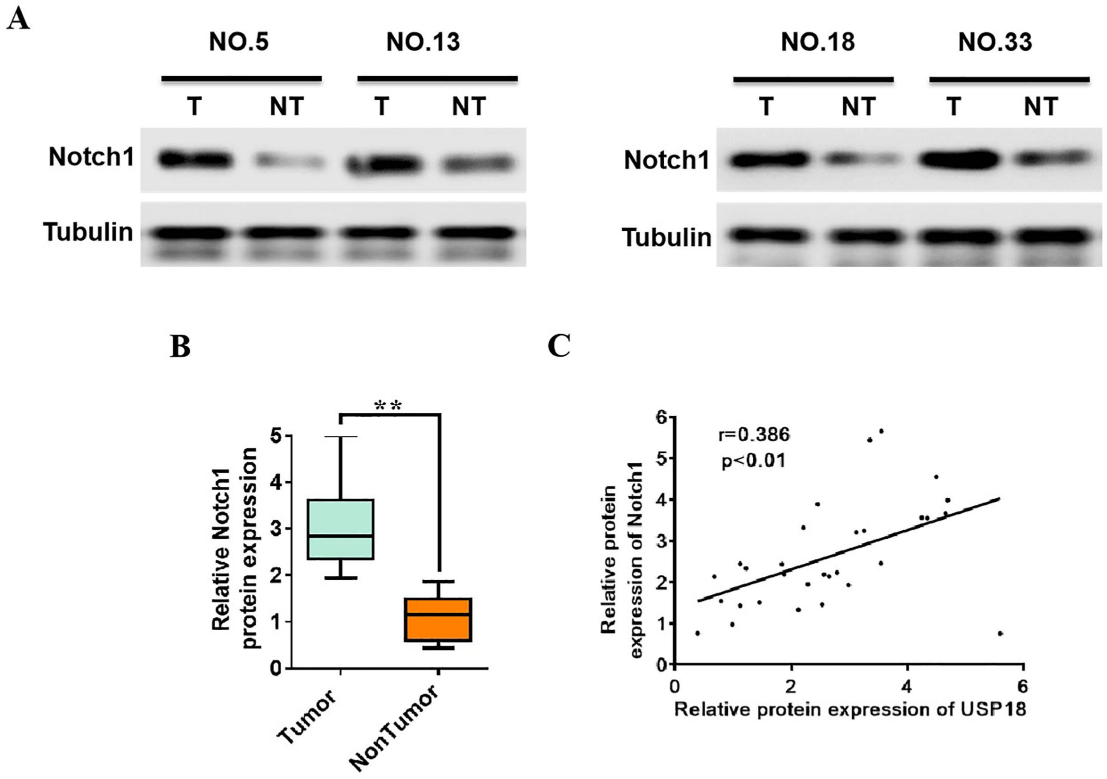

Supplementary Figure 5. (**A**, **B**), Determination and quantification of Notch1 protein levels in pancreatic cancer tissues and paired non-tumour tissues by western blotting. Tubulin served as a loading control. (**C**) Scatter plots of USP18 and Notch1 protein expression in pancreatic cancer.
