# Supplementary material for: Deubiquitinase USP18 promotes the progression of pancreatic cancer via enhancing the Notch1-c-Myc axis
Source: Aging (Albany NY). 2020 Oct 13;12(19):19273–92. doi: 10.18632/aging.103760 (PMC7732327; doi:10.18632/aging.103760)
Supplement: Supplementary Table 1 [file aging-12-103760-s002..pdf]

SUPPLEMENTARY TABLE

Supplementary Table 1. Summary of significantly differentially expressed genes in BxPC-3-shUSP18 compared with BxPC-3-shNC cells.

Gene name	Fold change	*P* value
**Increased expression**		
CEBPa	2.643	0.009
NFIA	2.457	0.002
SCARA5	3.392	0.007
SCN1A	2.673	0.002
VHL	2.752	0.044
SMOX	2.089	0.003
BDNF	2.511	0.034
KLF4	2.132	0.041
EDN2	2.365	0.004
HBXAP	2.592	0.024
PLAC8	2.355	0.041
PCDH7	2.037	0.035
PDK1	2.227	0.021
**Decreased expression**		
**Myc**	**5.381**	**0.014**
ROCK1	2.123	0.017
BZW1	2.123	0.022
UBR5	2.151	0.014
SEL1L	2.134	0.003
Cyclin D1	2.412	0.002
MDM2	2.453	0.003
ZEB1	2.418	0.002
ATG3	2.647	0.012
GLT25D2	2.221	0.027
UBD	2.144	0.012
LAPTM4A	2.111	0.022
